# *In vitro* activity of SF001: a next-generation polyene versus amphotericin B

**DOI:** 10.1128/aac.00322-25

**Published:** 2025-04-22

**Authors:** Roya Vahedi-Shahandashti, Cornelia Lass-Flörl

**Affiliations:** 1 Institute of Hygiene and Medical Microbiology, Medical University of Innsbruck 172142, Innsbruck, Tyrol, Austria; University Children's Hospital Münster, Münster, Germany

**Keywords:** antifungal resistance, novel antifungal agents, antifungal therapy, antifungal susceptibility testing, amphotericin B toxicity, invasive fungal infections (IFIs), amphotericin B resistance

## Abstract

SF001, a next-generation polyene drug, offers broad-spectrum fungicidal activity with less potential for toxicity than classic polyene amphotericin B (AmB). This study compared the *in vitro* activity of SF001 and amphotericin B against *Candida* and *Aspergillus* species. SF001 demonstrated activity comparable to AmB against *Candida* isolates (MIC_50/90_ of 0.25/1 and 0.5/0.5 mg/L, respectively). However, *Aspergillus* isolates exhibited higher susceptibility to SF001 than AmB (MIC_50/90_ of 0.5/1 and 1/4 mg/L, respectively), notably including AmB-resistant species.

## INTRODUCTION

Invasive fungal infections (IFIs) have increased in recent decades, largely driven by the rising number of immunosuppressed patients (1). IFIs are notoriously challenging to treat, often requiring systemic antifungal therapies, such as echinocandins, polyenes, and triazoles (2). Amphotericin B (AmB), a polyene antifungal, has long been a cornerstone in treating various systemic fungal infections for the past six decades, demonstrating strong clinical and pharmacological activity (3, 4). Despite the high toxicity of AmB limiting clinical use, it remains the gold standard for treating severe IFIs, where rapid response is crucial as is AmB’s broad spectrum of activity against yeasts and filamentous and dimorphic fungi (5, 6). The resistance rate of AmB is lower than that of other antifungals due to its fungicidal properties, along with the significant fitness cost associated with resistance (7, 8). Given AmB’s strengths, formulations like AmB lipid complex, liposomal AmB, and AmB colloidal dispersion were developed to reduce nephrotoxicity and infusion reactions (3). Still, the use of these formulations is limited, in part due to high rates of infusion-related events (9, 10), highlighting the unmet need for novel, safer options (11).

SF001, a next-generation polyene drug currently in clinical development, was rationally designed to mitigate toxicity (12). Its active moiety, Sfu-AM2-19 (formerly, AM-2-19) (12) has demonstrated potent, broad-spectrum fungicidal activity, including activity against *Aspergillus* strains resistant to AmB (12, 13). The current study aimed to compare the *in vitro* potency of SF001 and AmB (Amphotericin B from *Streptomyces* sp., Sigma-Aldrich, A4888) against representative clinical isolates of two common fungal genera, *Aspergillus* (*n* = 41) and *Candida* (*n* = 56), including AmB-resistant/non-wild-type strains. The isolates were tested according to the mould and yeast EUCAST guidelines (14, 15). The minimum inhibitory concentrations (MICs) of SF001 and AmB were determined using two criteria: for yeasts, the lowest concentration achieving 90% growth inhibition after 24 hours by spectrophotometry, and for molds, the lowest concentration showing no visible growth to the naked eye after 48 hours of incubation. *A. flavus* ATCC 204304, *C. krusei* ATCC 6258, and *C. parapsilosis* ATCC 22019 served as the quality control strains for each run of the assay (16). MIC ranges and MIC_50_ and MIC_90_ values (the concentrations that inhibited 50% and 90% of the isolates, respectively) were determined.

**Fig 1:**
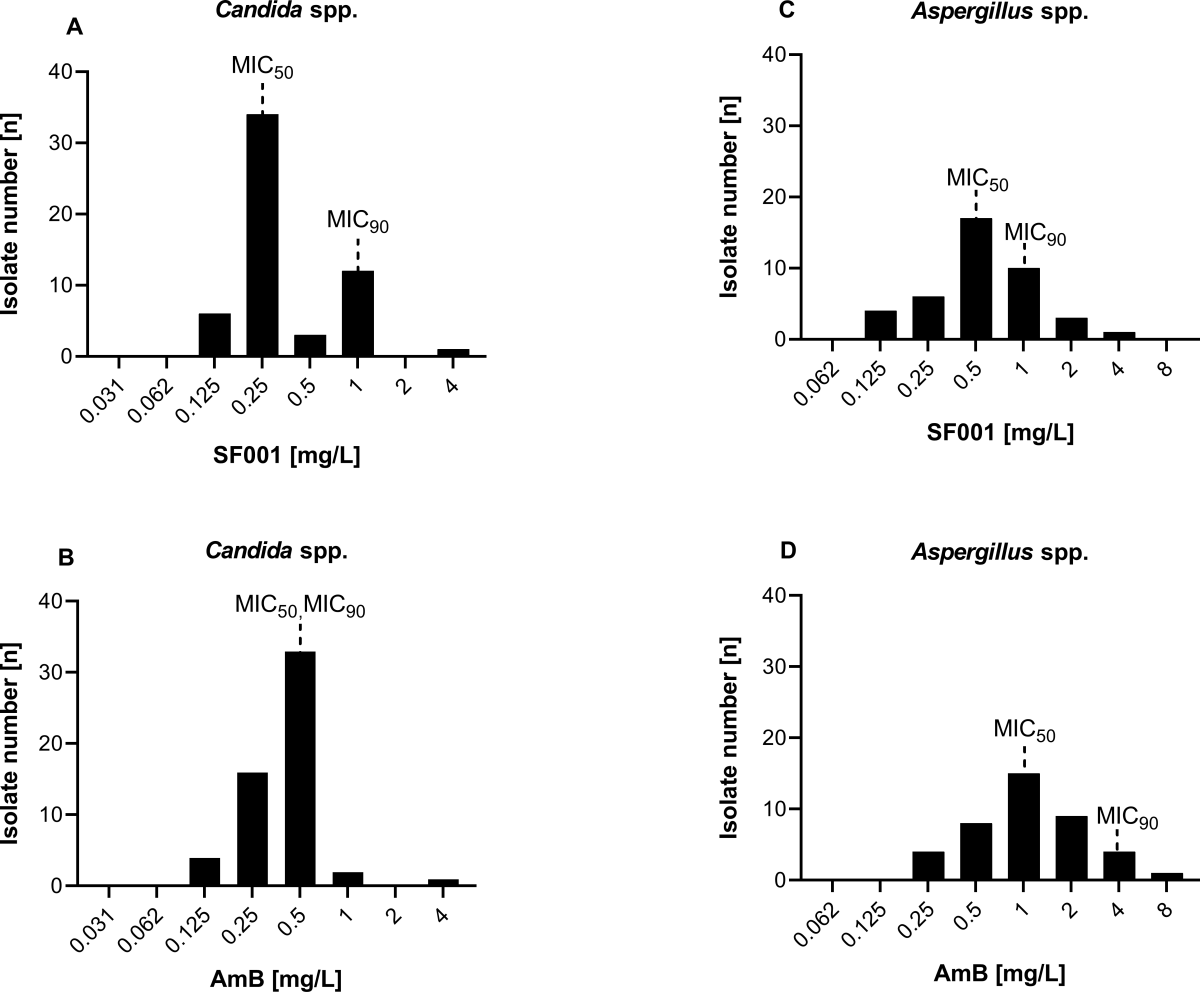
The distribution of minimum inhibitory concentrations of SF001 and amphotericin B against *Candida* species (A and B, respectively) and *Aspergillus* species (C and D, respectively). MIC_50_/MIC_90_ stand for concentrations inhibiting ≥50% and ≥90% of the tested isolates, respectively.

SF001 exhibited strong *in vitro* activity against both *Candida* and *Aspergillus* genera, with distinct variations in activity between the two (Fig. 1; Table 1). As shown in Fig. 1A and B, the MIC range of SF001 against *Candida* species was comparable to that of AmB (0.125 to 4 mg/L). The MIC_50_ and MIC_90_ values for SF001 were 0.25 and 1 mg/L, respectively, while for AmB, they were 0.5 mg/L (Fig. 1A and B). In contrast, against *Aspergillus* isolates, SF001 demonstrated a lower MIC range (0.125 to 4 mg/L) compared to that of AmB (0.25 to 8 mg/L), along with lower MIC_50_ and MIC_90_ values of 0.5 and 1 mg/L, versus 1 and 4 mg/L for AmB, respectively (Fig. 1C and D). Considering the geometric mean (GM) values, SF001 and AmB exhibited similar overall activity against all tested wild-type and AmB-resistant/non-wild-type *Candida* isolates (GM: 0.34 and 0.39 mg/L, respectively). In contrast, SF001 displayed greater activity against all tested *Aspergillus* species, with GM values of 0.54 mg/L for SF001 and 1.07 mg/L for AmB (Table 1). Although the overall GM MIC values of SF001 and AmB against *Candida* species were comparable, a species-specific analysis revealed subtle variations (Table 1). Species such as *C. glabrata*, *C. parapsilosis*, *C. tropicalis*, *C. dubliniensis*, and *C. inconspicua* exhibited a slight reduction in the GM MIC of SF001 compared to AmB. Conversely, other species, including *C. albicans*, *C. krusei*, *C. auris*, *C. lusitaniae*, and *C. guilliermondii*, showed higher GM MIC of SF001 compared to AmB (Table 1). Considering *Aspergillus* species separately (Table 1), SF001 demonstrated potent *in vitro* activity against all tested *Aspergillus* species, as reflected by lower GM MIC values, particularly for *A. terreus* (7, 17), *A. flavus* (17, 18), and *A. versicolor* (19, 20), which are commonly resistant to AmB. Interestingly, some *A. terreus* and *A. flavus* isolates classified as non-wild-type for AmB (with high MICs of 4–8 mg/L) showed much lower MICs (≤2 mg/L) when tested with SF001. If AmB EUCAST epidemiological cutoff values (ECOFFs) were applied to SF001, these isolates would be considered wild type. However, as SF001-specific breakpoints (BPs) and ECOFFs have yet to be established, caution is required when interpreting susceptibility categorization.

**TABLE 1:** Susceptibility profiles of SF001 and amphotericin B (AmB) against *Candida* and *Aspergillus* species, using EUCAST guidelines

Species	SF001 (mg/L)		AmB (mg/L)		AmB	
	MIC range	GM^*a*^ MIC	MIC range	GM^*a*^ MIC	BP^*b*^	ECOFF/TECOFF^*c*^
*C. albicans* (*n* = 6)	0.25–4	0.79	0.25–4	0.70	1	1
*C. glabrata* (*n* = 6)	0.25–0.5	0.28	0.5	0.5	1	1
*C. krusei* (*n* = 5)	0.25–1	0.65	0.5	0.5	1	1
*C. auris* (*n* = 5)	0.25–1	0.75	0.5	0.5	–^*d*^	2
*C. lusitaniae* (*n* = 5)	0.25	0.25	0.125–0.25	0.18	–	0.5
*C. guilliermondii* (*n* = 5)	0.125–1	0.32	0.125–0.5	0.25	–	0.5
*C. parapsilosis* (*n* = 5)	0.25	0.25	0.5	0.5	1	1
*C. tropicalis* (*n* = 5)	0.25	0.25	0.25–0.5	0.43	1	1
*C. dubliniensis* (*n* = 5)	0.125–0.25	0.21	0.125–0.5	0.25	1	0.25
*C. inconspicua* (*n* = 5)	0.125–0.25	0.14	0.25–0.5	0.28	–	–
*C. pararugosa* (*n* = 1)	0.25	–	0.5	–	–	–
*C. nivariensis* (*n* = 2)	0.25	–	0.5	–	–	–
*C. kefyr* (*n* = 1)	1	–	0.5	–	–	1
*Candida* species	0.125–4	0.34	0.125–4	0.39		
*A. fumigatus* (*n* = 6)	0.25–0.5	0.35	0.5–1	0.70	1	1
*A. flavus* (*n* = 6)	0.5–2	0.62	1–4	1.41	–	4
*A. niger* (*n* = 5)	0.125–0.25	0.14	0.25–0.5	0.37	1	0.5
*A. nidulans* (*n* = 6)	0.25–1	0.5	0.5–2	1	–	4
*A. lentulus* (*n* = 4)	1	1	2	2	–	–
*A. fumigatiaffinis* (*n* = 3)	0.5–4	–	1–4	–	–	–
*A. versicolor* (*n* = 4)	0.5–1	0.70	1–2	1.18	–	–
*A. terreus* (*n* = 7)	0.25–2	0.82	0.25–8	1.34	–	8
*Aspergillus* species	0.125–4	0.54	0.25–8	1.07		

^*a*^ MIC ranges and geometric mean (GM) values are reported only for species represented by at least four isolates.

^*b*^ BP, breakpoint (21).

^*c*^ ECOFF/TECOFF, EUCAST epidemiological cut-off values/tentative ECOFF (21).

^*d*^ – indicates that no ECOFF, TECOFF, or BP is available for the specific species.

The active moiety of SF001, Sfu-AM2-19, is a novel analog of AmB rationally designed to reduce toxicity by selectively extracting ergosterol from fungal membranes without binding to cholesterol in mammalian cells (12). This is achieved through C2′ epimerization and C16 amidation, enhancing ergosterol extraction while preventing cholesterol binding (12). The activity of SF001 in this study was consistent with previous findings by Maji et al. (12), which showed potent *in vitro* activity against fungal pathogens relatively resistant to liposomal AmB and frequently used azoles, i.e., isavuconazole, voriconazole, and posaconazole. However, the extent of activity varied among pathogens, with a minimal reduction in MIC for some strains (e.g., *C. krusei*) and a more substantial decrease in MICs for others (e.g., *A. fumigatus*), aligning with our findings. In addition, previous studies demonstrated SF001’s broad *in vivo* efficacy in candidiasis, aspergillosis, and mucormycosis mouse models, with dose-dependent reductions in fungal burden and reduced toxicity to human cells and mice (12, 13). SF001 demonstrates notable *in vitro* potency, particularly against common AmB-resistant and non-wild-type *Aspergillus* species, such as *A. terreus* and *A. flavus*, as well as less common species like *A. versicolor*. Its potent activity against resistant *Aspergillus* species underscores its potential to address the growing challenge of azole resistance—a significant limitation of current therapies—and the urgent need for new antifungals to combat escalating resistance and associated clinical failures (22–24). Similarly, the activity of SF001 against *Candida* species (Table 1) was comparable to AmB and is notable in the context of echinocandin- and fluconazole-resistant *Candia* species, which are also on the rise (23) and for which there are limited treatment options. SF001 safety data to date, combined with its potent fungicidal activity and ability to achieve pharmacokinetic/pharmacodynamic targets, positions SF001 as a strong candidate for further development (12, 13). Its effective *in vitro* activity, particularly against *Aspergillus* species, supports interest in further *in vivo* and clinical studies of SF001 to evaluate its potential role in treating IFIs.

## Data Availability

The authors confirm that all protocols are detailed within the article.
